# Continuous venovenous hemofiltration in severely burned patients with acute kidney injury: a cohort study

**DOI:** 10.1186/cc7801

**Published:** 2009-05-01

**Authors:** Kevin K Chung, Jonathan B Lundy, James R Matson, Evan M Renz, Christopher E White, Booker T King, David J Barillo, John A Jones, Leopoldo C Cancio, Lorne H Blackbourne, Steven E Wolf

**Affiliations:** 1 Burn Center, United States Army Institute of Surgical Research, 3400 Rawley E. Chambers Drive, Fort Sam Houston, TX 78234, USA; 2 Pediatric Acute Care Associates of North Texas, PA, 7777 Forest Lane, D569, Dallas, TX 75230, USA; 3 Department of Surgery, UT Health Science Center at San Antonio, 7703 Floyd Curl Drive, San Antonio, TX 78229, USA

## Abstract

**Introduction:**

Acute kidney injury (AKI) is a common and devastating complication in critically ill burn patients with mortality reported to be between 80 and 100%. We aimed to determine the effect on mortality of early application of continuous venovenous hemofiltration (CVVH) in severely burned patients with AKI admitted to our burn intensive care unit (BICU).

**Methods:**

We performed a retrospective cohort study comparing a population of patients managed with early and aggressive CVVH compared with historical controls managed conservatively before the availability of CVVH. Patients with total body surface area (TBSA) burns of more than 40% and AKI were treated with early CVVH and their outcomes compared with a group of historical controls.

**Results:**

Overall, the 28-day mortality was significantly lower in the CVVH arm (n = 29) compared with controls (n = 28) (38% vs. 71%, *P *= 0.011) as was the in-hospital mortality (62% vs. 86%, *P *= 0.04). In a subgroup of patients in shock, a dramatic reduction in the pressor requirement was seen after 24 and 48 hours of treatment. Compared with controls (n = 19), significantly fewer patients in the CVVH group (n = 21) required vasopressors at 24 hours (100% vs 43%, *P *< 0.0001) and at 48 hours (94% vs 24%, *P *< 0.0001). In those with acute lung injury (ALI)/acute respiratory distress syndrome (ARDS), there was a significant increase from baseline in the partial pressure of arterial oxygen (PaO_2_) to fraction of inspired oxygen (FiO_2_) ratio at 24 hours in the CVVH group (n = 16, 174 ± 78 to 327 ± 122, *P *= 0.003) but not the control group (n = 20, 186 ± 64 to 207 ± 131, *P *= 0.98).

**Conclusions:**

The application of CVVH in adult patients with severe burns and AKI was associated with a decrease in 28-day and hospital mortality when compared with a historical control group, which largely did not receive any form of renal replacement. Clinical improvements were realized in the subgroups of patients with shock and ALI/ARDS. A randomized controlled trial comparing early CVVH to standard care in this high-risk population is planned.

## Introduction

Acute kidney injury (AKI) is a common and devastating complication in critically ill burn patients with an incidence reported to be as high as 30% and mortality reported to be between 80 and 100% [1-3]. This AKI-associated mortality appears to be substantially higher in the severely burned than the general ICU population, recently reported to be 60% [4]. Regardless, much as in other critically ill populations, mortality associated with AKI has not improved in this high-risk population over time despite advances in burn care and renal replacement techniques. The US Army Burn Center at the United States Army Institute of Surgical Research, San Antonio, Texas, is the sole burn treatment facility in the Department of Defense serving active duty personnel stationed around the world. In addition, the Army Burn Center serves as the regional burn center for all of South Texas, covering an area of 80,000 square miles with a population approaching five million.

Prior to November 2005, only conventional intermittent hemodialysis (IHD) was available as a renal replacement modality for those who developed AKI. Strict traditional criteria such as profound hyperkalemia, symptomatic uremia, refractory fluid overload, and refractory acidosis were used to determine the need for IHD. In addition, many patients were considered to be poor candidates for IHD because of the presence of hemodynamic instability and thus not offered therapy. In this population, continuous venovenous hemofiltration (CVVH) seemed to be an attractive and necessary modality because it is well tolerated by hemodynamically unstable patients and allows for optimal metabolic management. Thus, an intensivist-driven CVVH program was developed for our Burn Intensive Care Unit (BICU). We hypothesized that early intervention with CVVH in severely burned patients with AKI would result in better outcomes when compared with the traditional approach. Data on its impact on military casualties has previously been reported [5]. This study was conducted to expand the analysis to include all civilian patients treated in our BICU.

## Materials and methods

### Patients

After obtaining approval from our hospital Institutional Review Board, we conducted a retrospective cohort study. Consecutive severely burned patients, with more than 40% total body surface area (TBSA) with AKI who were initiated on CVVH between November 2005 and August 2007 were compared with closely matched historical controls identified prior to the availability of CVVH in the BICU (March 2003 to November 2005). Inclusion criteria for the control group included a diagnosis of acute renal failure, an injury severity score (ISS) of more than 25 and a burn size of more than 40% TBSA. First a list of all patients with a diagnosis of 'renal failure' or 'renal insufficiency' appearing on their electronic medical record was cross-matched with a list of all patients with an ISS of more than 25. Second, a query of all those in whom nephrology consultation was requested was performed and those patients not on the initial query were added. Finally, a query of all patients with a diagnosis of more than 40% TBSA was generated and a chart review of all patients not on the initial two queries performed in order to identify patients meeting the diagnosis of AKI (Acute Kidney Injury Network (AKIN) stage 2 or greater) that did not have it documented on their list of diagnoses. In both groups, those patients who were moribund (who lived less than 12 hours) or had the diagnosis of chronic renal insufficiency were excluded. We recorded baseline demographic, laboratory, and hemodynamic parameters. In-hospital mortality and 28-day mortality were compared.

### Definitions

During the period after November 2005 our staff intensivists utilized the RIFLE (Risk, Injury, Failure, Loss, End-stage kidney disease) classification to categorize the severity of AKI [6]. The AKIN classification system has since been developed to replace the RIFLE classification [7]. For this analysis, in both groups, we have applied the AKIN criteria *post hoc*. This system defines stage 1 as an increase in serum creatinine by 0.3 mg/dl or an increase to more than or equal to 1.5 to 2-fold from baseline or a urine output of less than 0.5 ml/kg/hour for six hours. Stage 2 is defined as an increase in creatinine by two to three-fold or a urine output less than 0.5 ml/kg/hour for 12 hours. Stage 3 is defined as an increase in serum creatinine by more than three-fold or urine output less than 0.3 ml/kg/hour for 24 hours or anuria for 12 hours [7]. Sepsis and septic shock are defined according to previously published guidelines [8]. Shock was defined by the presence of hypotension not responsive to fluid (crystalloid or colloid) resuscitation that required vasopressor therapy. Acute lung injury (ALI) and acute respiratory distress syndrome (ARDS) are also defined according to previously published guidelines [9]. In the CVVH group, the index day (T0) from which all subsequent data were recorded was defined as the day CVVH was initiated. In the control arm, T0 was defined as the day the patient met the diagnosis of AKI (AKIN stage 2) with shock or AKI (AKIN stage 3) without shock.

### Interventions

Patients treated during the period prior to the availability of CVVH in the BICU received standard care for acute renal failure which included: fluid resuscitation, minimization of nephrotoxic agents, and utilization of hemodialysis if classic indications were met. These indications included: refractory acidosis, electrolyte abnormalities, symptomatic fluid overload not responsive to conservative interventions, and intoxication with a dialyzable agent. Consultation from the nephrology service was requested by the BICU intensivists when deemed necessary based on the severity of the dysfunction.

The intensivists that staff the BICU have prescribed and supervised the use of CVVH since the program was started. During the study period, patients were treated via CVVH with the Prismaflex system (Gambro, Lund, Sweden) using a polyarylethersulfone (PAES) filter, which has a nominal molecular weight cut-off of 50 kDa and a surface area of 1.4 m^2^. Patients were each identified as candidates for therapy if they were AKIN stage 3 or AKIN stage 2 with shock. Typically the replacement fluid was infused both pre- and post-filter divided equally. The initial prescribed dose varied from 30 to 120 mL/kg/hour based on a compilation of various clinical parameters to include catabolic state, degree of solute and electrolyte imbalance, and presence of shock. Once patients were thought to be at steady state (24 to 36 hours) the dose was decreased to a 'maintenance dose' of 20 to 35 mL/kg/hour for the duration of therapy. Therapy was discontinued when patients exhibited evidence of renal recovery with greater than 0.5 ml/kg/hour urine volume for more than 24 hours. The study group also received the other measures considered part of standard care in patients with renal dysfunction including: fluid resuscitation, and avoidance of nephrotoxic substances.

### Statistical analysis

The primary outcome measures of interest were 28-day mortality and in-hospital mortality. Data were analyzed using SPSS version 16.0 (SPSS Inc., Chicago, IL, USA). Comparisons were made between the CVVH group and control group. Data are presented as mean ± standard deviation unless specified as median with interquartile range (IQR, Q1 to Q3). A multiple logistic regression analysis was performed for the total number of patients in both groups to determine the effect on the risk of death of the following variables: age, percentage TBSA, percentage full-thickness TBSA, inhalation injury, ISS, multiple organ dysfunction score (MODS), sequential organ failure assessment (SOFA), acute physiology and chronic health evaluation (APACHE II), AKIN stage, and treatment group. Continuous variables were compared via paired student t-test or Wilcoxon rank-sum test. Chi-square testing was used to compare categorical variables. All testing was two-tailed, with *P *< 0.05 considered significant. Kaplan-Meier estimate of survival was constructed to compare one-year survival between the CVVH group and the control group via stratified log-rank test.

## Results

After the start of our CVVH program in November 2005, 361 patients were admitted to the BICU during a 22-month period. Of these, 38 consecutive patients were treated using CVVH with 29 meeting our original inclusion criteria. Nine patients, four who survived to discharge, were excluded for the following reasons: isolated inhalation injury (n = 1), preexisting chronic renal insufficiency (n = 4), percentage TBSA less than 40% (n = 1), and non-thermal injury (n = 3). Prior to the start of our program, 486 patients were admitted to the burn ICU during a 32-month period. Of these, 42 patients were identified by our multi-step query for renal failure. Fourteen patients, 10 who survived to discharge, were excluded for the following reasons: preexisting chronic renal insufficiency (n = 3), burn size of less than 40% TBSA (n = 6), non-thermal injury (n = 4), and nephrology consultation for lithium toxicity (n = 1).

In all, 28 patients met our original inclusion criteria. Of these, 15 patients were evaluated by nephrology, with two being placed on IHD. Table 1 lists patient demographics at the time of the diagnosis of AKI, nephrology consultation, or the initiation of CVVH (T0). No significant differences were detected between the two groups with respect to any of the demographic variables, burn size, severity of illness scores, AKIN stage, initial serum blood urea nitrogen (BUN) or creatinine, presence of shock, and ALI/ARDS. The CVVH group was initiated on therapy (T0) at a median of 9 (IQR = 1 to 21) days after admission for AKI. The control group, by comparison, was diagnosed with AKI (T0) at a median of 19 (IQR = 8 to 25) days after admission (*P *= 0.32). 'Early AKI', as defined as the presence of AKI within 14 days from time of admission, occurred in 62% of patients in the CVVH group and 46% of patients in the control group (*P *= 0.24). Patients in the CVVH group were initially prescribed a mean hemofiltration dose of 57 ± 19 ml/kg/hour. The mean duration of treatment was 5.6 ± 4.1 days.

**Table 1:** Patient demographics

	Control group (n = 28)	CVVH group (n = 29)	*P *value
Age	38 ± 18	27 ± 8	0.06
Percentage TBSA	58 ± 18	64 ± 18	0.21
Percentage full thickness	43 ± 27	49 ± 22	0.37
Inhalation injury	61%	41%	0.14
ISS	36 ± 14	35 ± 13	0.80
APACHE II#	36 ± 8	35 ± 5	0.76
MODS#	13 ± 4	12 ± 3	0.53
SOFA#	13 ± 4	13 ± 3	0.87
AKIN stage 3	71%	70%	0.84
BUN (mg/dl)*	58 ± 26	46 ± 24	0.07
Creatinine (mg/dl)*	2.8 ± 1	2.9 ± 2	0.88
Hospital day of T0	23 ± 26	17 ± 24	0.21
Septic shock	43%	42%	0.94
Shock (all cause)	68%	72%	0.50
ALI/ARDS	71%	55%	0.20

All values calculated as means ± standard deviation. **# **Calculated at T0. *Measured on T0 – day of continuous renal replacement therapy initiation, diagnosis of renal failure or nephrology consultation.

AKIN = acute kidney injury network; ALI = acute lung injury; APACHE = acute physiology and chronic health evaluation; ARDS = acute respiratory distress syndrome; BUN = blood urea nitrogen; CVVH = continuous venovenous hemofiltration; ISS = injury severity score; MODS = multiple organ dysfunction score; SOFA = sequential organ failure assessment; TBSA = total body surface area.

**Figure 1:**
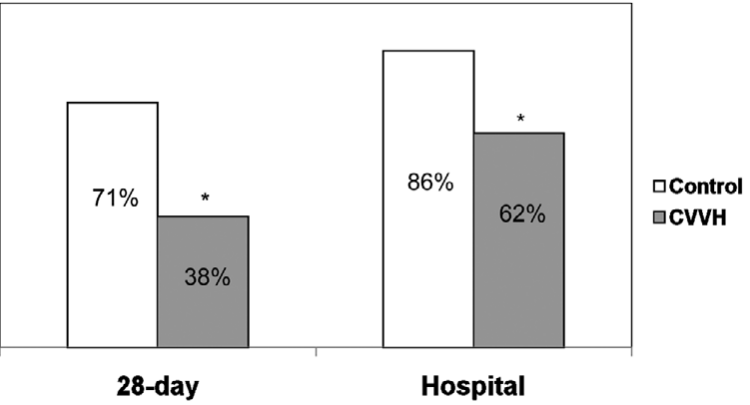
A comparison of 28-day and hospital mortality between the two groups. * *P *< 0.05. CVVH = continuous venovenous hemofiltration.

**Figure 2:**
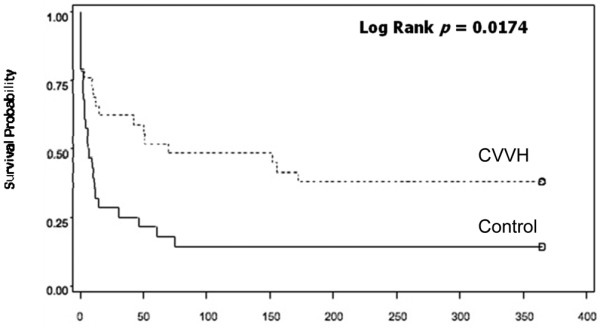
Kaplan-Meier estimates of survival between the two groups. Continuous venovenous hemofiltration (CVVH) was associated with a significantly higher rate of survival out to over one year.

Overall, the 28-day mortality was significantly lower in the CVVH arm compared with controls (38% vs. 71%, *P *= 0.011) as was the in-hospital mortality (62% vs. 86%, *P *= 0.04; Figure 1). The Kaplain-Meier survival curve demonstrated a significantly higher rate of survival in the CVVH group compared with the control group extending from T0 to over 350 days (Figure 2). All surviving patients treated with CVVH had return of renal function to baseline values at the time of discharge from the hospital. Three survivors in the control group had renal recovery, although the two patients who underwent IHD died.

**Figure 3:**
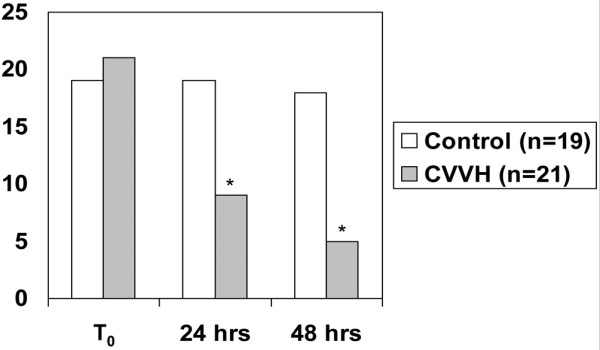
Subgroup of patients in shock. A comparison between the umber of patients on vasopressors at T_0_, 24 and 48 hours. * *P *< 0.05 both compared with baseline and between groups. CVVH = continuous venovenous hemofiltration.

A subgroup of patients in shock was analyzed. No significant differences were detected between the two groups with respect to multiple baseline physiologic parameters to include heart rate, mean arterial pressure, central venous pressure, and lactate (Table 2). Variability was detected with respect to the type and dose of vasopressors used between the two groups. Regardless, when compared with the control group, significantly fewer patients in the CVVH group required vasopressors at 24 hours (100% vs 43%, *P *< 0.0001) and at 48 hours (94% vs 24%, *P *< 0.001; Figure 3). There was no significant difference in the number of patients receiving stress dose steroids between the two groups.

**Table 2:** Shock patient comparison (at T0)

	Control group (n = 19)	CVVH group (n = 21)	*P *value
Heart rate	114 ± 20	121 ± 14	0.18
MAP (mmHg)	65 ± 13	61 ± 8	0.25
CVP (cmH_2_O)	18 ± 6	19 ± 8	0.85
Norepinephrine dose (μg/min)	4.2 ± 4.3	10.0 ± 12.6	0.06
Dopamine dose (μg/kg/min)	1.8 ± 3.6	0	< 0.0001
Vasopressin dose (units/min)	0.016 ± 0.020	0.038 ± 0.009	< 0.0001
Neosynephrine dose (μg/min)	4.9 ± 21.3	0	< 0.0001
Dobutamine dose (μg/kg/min)	0.26 ± 1.1	7.6 ± 7.8	< 0.001
Mean lactate (mmol/L)	2.9 ± 1.8	5.1 ± 4.2	0.12
Steroids	53%	33%	0.22

All values reported as means ± standard deviation except for steroids.

CVP = central venous pressure; CVVH = continuous venovenous hemofiltration; MAP = mean arterial pressure.

**Figure 4:**
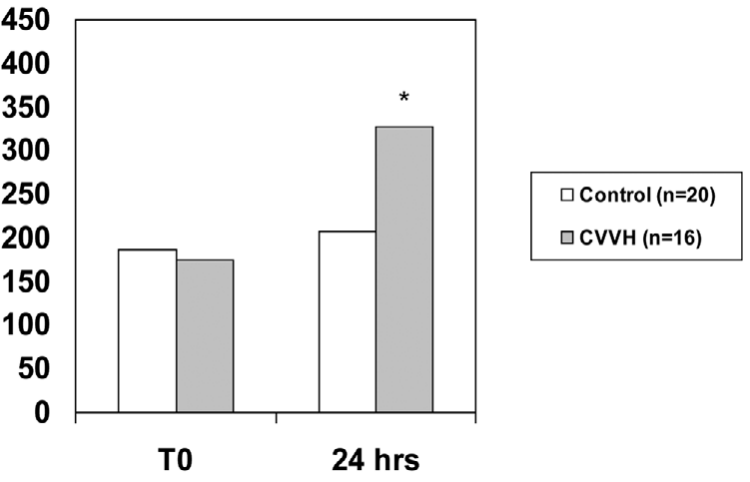
Subgroup of patients with acute lung injury/acute respiratory distress syndrome. Partial pressure of arterial oxygen/fraction of inspired oxygen ratio in patients with acute lung injury/acute respiratory distress syndrome at T_0 _and 24 hours. * *P *< 0.05 compared both from baseline (T0) and between groups.

In the subgroup of patients with ALI/ARDS the ratio of partial pressure of arterial oxygen (PaO_2_) to fraction of inspired oxygen (FiO_2_) increased significantly between T0 and T24 in the CVVH group (174 ± 78 to 327 ± 122, *P *= 0.003), but did not change in the control group (186 ± 64 to 207 ± 131, *P *= 0.98; Figure 4). No significant physiologic differences were detected in this subgroup (Table 3). The PaO_2_/FiO_2 _ratio improved in the CVVH arm independent of volume status.

**Table 3:** Physiologic data for the subgroup of patients with ALI/ARDS

	Control group (T0)	Control group (T+24)	CVVH group (T0)	CVVH group (T+24)
MAP (mmHg)	65 ± 13	72 ± 13	61 ± 8	72 ± 6
CVP (cmH_2_O)	18 ± 5	22 ± 5	18 ± 6	19 ± 4
Total fluids (liters)	13 ± 4*	13 ± 5	13 ± 5*	11 ± 5
Urine output (ml)	1002 ± 657*	942 ± 743	978 ± 754*	688 ± 396
Diuretic use (%)	5*	25	6*	0
Weight (kg)	84 ± 8	85 ± 7	84 ± 7	84 ± 6

Control group (n = 20), CVVH (continuous venovenous hemofiltration) group (n = 16).* Values reported represent the totals in the 24-hour period prior to T0. For all comparisons (*P *= ns).

CVP = central venous pressure; MAP = mean arterial pressure.

On multiple logistic regression analysis, only SOFA score was determined to be predictive of death at 28 days and in-hospital (odds ratio = 1.43, 95% confidence interval = 1.14 to 1.78). There were significant correlations between vasopressor requirement at 24 (Phi coefficient = 0.302, *P *= 0.035) and 48 hours (Phi coefficient = 0.450, *P *= 0.003) with 28-day mortality. In the control group there was a significant correlation between the presence of ALI/ARDS and 28-day mortality (Phi coefficient = 0.475, *P *= 0.012). This correlation did not exist in the CVVH arm.

## Discussion

Our previous study demonstrated that aggressive intervention with CVVH in critically ill burned military patients with a high risk of death was associated with an improved survival when compared with a closely match historical cohort [5]. Decreases in 28-day and in-hospital mortality were sustained when an additional 23 civilian patients (12 in the control group and 11 in the CVVH group) were added to this analysis [5]. The present expanded analysis serves to underscore our previous observation that aggressive application of CVVH may be beneficial in the critically burned population who develop AKI. Furthermore, we are able to shed some light on specific subgroups of patients (shock and ALI/ARDS) who may benefit from our treatment technique.

Our findings highlight a few important points. The most compelling is the unreasonably high mortality (86% in hospital) seen in critically ill burn patients who develop AKI in our carefully selected historical control patients. This mortality closely matches that seen in previous studies as the reported mortality in severely burned patients with AKI has exceeded 80% historically [1-3,5,10-12]. Therefore, this group appears valid. In severely burned patients, AKI is associated with a much higher mortality than what has been reported for the overall ICU population [4]. 'Early AKI' may have a different pathophysiologic mechanism, as well as prognosis, than AKI occurring later in the hospital course. Overall, 54% of the patients developed 'early AKI', defined as AKI occurring within 14 days of admission. However, there was a trend towards a higher incidence of 'early AKI' in the CVVH group vs the control group (62% vs 46%, *P *= 0.24).

The second point to emphasize is that in this high-risk population, our study suggests that aggressive application of CVVH is superior to a traditional conservative approach. In the control group, nephrology consultation was requested in 15 out of 28 patients. Of these patients, only two were placed on IHD. The others, with or without nephrology consultation, were either thought to be too hemodynamically unstable to tolerate IHD, the only modality available at that time, or did not meet the traditional criteria for therapy. Thus, our study essentially compares a cohort of patients (the CVVH group) who received a renal replacement therapy versus a cohort who largely did not.

One variable that may confound the difference in outcome may be how we have defined 'standard' care received by the patients in the historical cohort. It is unclear what impact, if any, a more aggressive approach with the application of earlier IHD or another intermitted technique such as sustained low-efficiency dialysis (SLED) would have had on the mortality rate in this group [13]. As part of 'standard' practice, the need for acute renal replacement therapy was primarily guided by absolute indications (i.e. refractory fluid overload, refractory acidosis, symptomatic uremia). If a patient did not meet one of these indications they were not placed on therapy. The few patients who did meet one or more of these criteria (three patients had BUN of more than 100) were thought to be too hemodynamically unstable for therapy. These patients may have been candidates for SLED. Unfortunately, this capability did not exist at our facility.

In our patients, our standard practice was to initiate CVVH for either the diagnosis of AKI or AKI in the presence of shock. In the eight patients with isolated AKI, the average BUN was 61 ± 28 mg/dl. *Post hoc*, two patients were classified as AKIN stage 2 and six patients were AKIN stage 3. In those with AKI and shock (n = 21), the average BUN at time of initiation of CVVH was 41 ± 21 mg/dl. Of these patients, 13 were classified as AKIN stage 3, six as stage 2, and two as stage 1. By any definition, this would be considered early application of renal replacement and this strategy may have played a significant role in conferring a survival benefit in the treatment group.

Our Kaplan Meier survival curve (Figure 2) illustrates that a significant number of patients in the control group died within 14 days from time of diagnosis (19 of 28 patients). Of these, nine died within two days. This suggests that most burn patients who develop AKI die before meeting these arbitrary criteria and that perhaps CVVH may be a lifesaving intervention in these patients. Bouman and colleagues demonstrated in a prospective randomized study (n = 106) that early initiation of hemofiltration did not result in a survival benefit [14]. Survival rates in their study approached or exceeded 70% in the three treatment groups evaluated. This is distinctly higher when compared with other studies (18 to 58%) [3,15,16]. Thus it is unclear if these findings can be extrapolated to the burn population, with historical survival rates of less than 30% [1-3,11]. Furthermore, all three groups in this study received some form of renal replacement in the course of their hospitalization. Clearly variation in practice exists within the field of nephrology and a more aggressive 'standard' approach with the application of any renal replacement modality may have had some impact [17-19]. Nevertheless, it appears that 'something is better than nothing'. It is likely that some renal replacement, in any population, is better than no renal replacement. To our knowledge, no study to date has prospectively tested this assumption.

A third point to emphasize is that our CVVH technique appears to have had extra-renal effects in these patients with the reversal of shock and improvement of ARDS. Our approach utilized primarily a hemofiltration-based therapy (CVVH) at a relatively high average prescribed dose (57.1 ± 18.9 ml/kg/hour). CVVH brings the theoretical benefit 'middle-molecule' elimination via convective clearance. Burn injury complicated by inflammatory states such as circulatory shock may benefit from higher doses of hemofiltration providing immunomodulary effects. In the subgroups of patients with shock, treatment effect is strongly suggested as the majority of patients are off pressors by 48 hours. Significant correlations between pressor requirement at 24 (Phi coefficient = 0.302, *P *= 0.035) and 48 hours (Phi coefficient = 0.450, *P *= 0.003) with 28-day mortality were noted. Additionally, the PaO_2_/FiO_2 _ratio is significantly improved compared with historical controls (Figure 4) independent of volume status. It is interesting to note that significant correlation was noted between the presence of ALI/ARDS and 28-day mortality (Phi coefficient = 0.475, *P *= 0.012) in the control group. This correlation did not exist in the CVVH arm, suggesting that CVVH may have altered the course of the disease. We are not the only ones to make this observation. Piccinni and colleagues reported strikingly similar results as they described an improvement in hemodynamics, gas exchange, and 28-day survival compared with historical controls after the institution of early, isovolemic hemofiltration for the treatment of oliguric patients with septic shock [20]. The 28-day survival of 55% was significantly higher than in the historical control arm (27%, *P *< 0.05). The authors hypothesized that early, high-volume hemofiltration may non-specifically affect mediators (both pro-inflammatory and anti-inflammatory) and improve outcomes by modulating both early, multiple organ dysfunction due to systemic inflammation and allowing for increased immunocompetence later in the course of sepsis. Others have reported the safety and potential efficacy of very high doses of hemofiltration in other single center prospective studies using a technique of short-term, high-volume hemofiltration [21,22].

It is important to note that the Veterans Affairs/National Institute of Health (VA/NIH) Acute Renal Failure Trial Network recently demonstrated no difference in 60-day mortality between intensive and less-intensive renal replacement therapy strategies in ICU patients with AKI in a large multi-center study [17]. The intensive therapy arm received a delivered dose of 35.8 ± 6.4 mL/kg/hour of continuous venovenous hemodiafiltration (CVVHDF) or an average of 5.4 sessions of IHD or sustained low-efficiency dialysis per week. The less-intensive group received a delivered dose of CVVHDF of 22.0 ± 6.1 mL/kg/hour or an average of three treatment sessions of IHD or SLED. Perhaps either approach applied in our treatment arm would have had the same survival benefit.

One must be appropriately cautious in extrapolating these findings to the burn population for two reasons. The vast majority of the VA/NIH trial did not involve burn patients. Burn patients have been characterized as being highly catabolic with coexisting complex fluid, electrolyte and acid-base management problems that exceed those in most critically ill patients in other settings [23,24]. Additionally, the majority of our patients had shock or had developed ALI/ARDS at baseline. It is unclear if the doses used in the VA/NIH study would be adequate to result in any extra-renal effects. In the CVVH group the initial prescribed dose was variable based on hemodynamic compromise and perceived metabolic stress at the time of initiation. Thus the prescribed dose varied from 30 to 120 mL/kg/hour. Those patients in shock at the time of initiation were prescribed a significantly higher dose of therapy (n = 21, 63 ± 20 mL/kg/hour) than those who were not in shock (n = 8, 46 ± 11 mL/kg/hour, *P *= 0.008). Both these prescribed doses are substantially higher than the 'high-dose' group in the VA/NIH trial.

The results of this study must be interpreted with caution. Several limitations to our study exist that are inherent to a retrospective study design. One could argue the potential for lead-time bias as the presence of such a capability could encourage earlier detection of AKI and lead to the treatment of those who would have otherwise performed well without renal replacement. In both groups, severity AKI was stratified via the AKIN staging criteria *post hoc*. Thus, it is possible that some were missed. The control group was identified by cross matching our trauma database for the diagnosis of renal failure with an ISS of more than 25, identification of all patients who were admitted with a more than 40% TBSA burn, and a list of all patients evaluated by nephrology during that time period. We made a concerted effort to capture as many patients as possible during this time period. The two groups appear very closely matched when comparing all measures of illness severity. However, we cannot overlook the fact that the trend for age and the incidence of inhalation injury were both higher in the control group. This may have contributed to bias in favor of the CVVH group. Additionally, there was a trend for the time of diagnosis relative to admission (T0), being earlier in the CVVH arm compared with the control arm. This further contributes to the lead-time bias in favor of the CVVH group.

In the four-year period, we must also assume that other aspects of care may have changed. Practice variation is evident in the types of vasopressors that were used in the patients in shock. Dopamine and neosynephrine were used more frequently in the control period, while vasopressin, dobutamine, and norepinephrine were preferred during the CVVH period. Additionally, a medical intensivist joined the BICU staff five months prior to the initiation of the CVVH program and helped standardize various ICU practices by instituting ICU-specific protocols to include sedation and analgesia, and transfusion guidelines. It is difficult to assess the possible confounding effect these changes may have had on patient survival. During the time period evaluated, however, surgical burn staff turn-over was minimal and surgical and wound care techniques remained the same.

## Conclusions

In a retrospective cohort study, when compared with conservative management, treatment of AKI in severe burns with a high ultrafiltrate dose was associated with a reduced vasopressor requirement, improved lung function, and a lower mortality rate. Aggressive application of CVVH may have a role in the treatment and prevention of extra-renal complications of AKI, burn shock, or septic shock. A fully funded randomized, multi-center prospective clinical trial addressing the application of CVVH in severely burned patients with septic shock and mild AKI is currently being planned.

## Key messages

- Patients with severe burns who develop AKI have a high rate of mortality.

- Application of CVVH is associated with better survival in our patients when compared with a traditional treatment approach.

- Improvement in survival may be related to the reversal of shock and/or the improvement in lung function.

- A prospective randomized clinical trial is needed and planned.

## Abbreviations

AKI: acute kidney injury; AKIN: acute kidney injury network; ALI: acute lung injury; APACHE: acute physiology and chronic health evaluation; ARDS: acute respiratory distress syndrome; BICU: burn intensive care unit; BUN: blood urea nitrogen; CVVH: continuous venovenous hemofiltration; CVVHDF: continuous venovenous hemodiafiltration; ESRD: end-stage renal disease; FiO_2_: fraction of inspired oxygen; IHD: intermittent hemodialysis; IQR: interquartile range; ISS: injury severity score; MODS: multiple organ dysfunction score; NIH: National Institute of Health; PAES: polyarylethersulfone; PaO_2_: partial pressure of arterial oxygen; SLED: sustained low-efficiency dialysis; SOFA: sequential organ failure assessment; TBSA: total body surface area; VA: Veterans Affairs.

## Competing interests

The authors declare that they have no competing interests.

## Authors' contributions

KKC was involved in study conception, design, data acquisition, analysis, and manuscript drafting. JBL was involved in data acquisition and manuscript drafting. JRM was involved in editing and revising the manuscript. EMR was involved in study conception and design. CEW was involved in study conception, design, and data acquisition. BTK was involved in editing and revising. DJB was involved in study conception and revising. JAJ was involved in statistical analysis and manuscript drafting. LCC was involved in study conception and revising. LHB was involved in editing and final approval of the manuscript. SEW was involved in study conception, data analysis, manuscript editing and supervision of the research group.

## Authors' information

KKC (medical intensivist) is the Medical Director of the burn ICU. He is also the Director of the CVVH program. JBL, BTK, CEW, DJB, and LCC are burn/trauma surgeons. JRM is a pediatric nephrologist and president and CEO of Immunocept LLC. SEW (burn surgeon) is the former Burn Director of the US Army Burn Center and current director of research. He is also the Editor-in-Chief of Burns. EMR is a burn/trauma surgeon and also the current director of the US Army Burn Center. LHB is the commander of the US Army Institute of Surgical Research.
